# Improved precision of QTL mapping using a nonlinear Bayesian method in a multi-breed population leads to greater accuracy of across-breed genomic predictions

**DOI:** 10.1186/s12711-014-0074-4

**Published:** 2015-04-17

**Authors:** Kathryn E Kemper, Coralie M Reich, Philip J Bowman, Christy J vander Jagt, Amanda J Chamberlain, Brett A Mason, Benjamin J Hayes, Michael E Goddard

**Affiliations:** Faculty of Veterinary and Agricultural Sciences, University of Melbourne, Parkville, 3052 Australia; Department of Environment and Primary Industries, AgriBio, Bundoora, 3086 Australia; La Trobe University, Bundoora, 3086 Australia; Dairy Futures Co-operative Research Centre, Bundoora, 3086 Australia

## Abstract

**Background:**

Genomic selection is increasingly widely practised, particularly in dairy cattle. However, the accuracy of current predictions using GBLUP (genomic best linear unbiased prediction) decays rapidly across generations, and also as selection candidates become less related to the reference population. This is likely caused by the effects of causative mutations being dispersed across many SNPs (single nucleotide polymorphisms) that span large genomic intervals. In this paper, we hypothesise that the use of a nonlinear method (BayesR), combined with a multi-breed (Holstein/Jersey) reference population will map causative mutations with more precision than GBLUP and this, in turn, will increase the accuracy of genomic predictions for selection candidates that are less related to the reference animals.

**Results:**

BayesR improved the across-breed prediction accuracy for Australian Red dairy cattle for five milk yield and composition traits by an average of 7% over the GBLUP approach (Australian Red animals were not included in the reference population). Using the multi-breed reference population with BayesR improved accuracy of prediction in Australian Red cattle by 2 – 5% compared to using BayesR with a single breed reference population. Inclusion of 8478 Holstein and 3917 Jersey cows in the reference population improved accuracy of predictions for these breeds by 4 and 5%. However, predictions for Holstein and Jersey cattle were similar using within-breed and multi-breed reference populations. We propose that the improvement in across-breed prediction achieved by BayesR with the multi-breed reference population is due to more precise mapping of quantitative trait loci (QTL), which was demonstrated for several regions. New candidate genes with functional links to milk synthesis were identified using differential gene expression in the mammary gland.

**Conclusions:**

QTL detection and genomic prediction are usually considered independently but persistence of genomic prediction accuracies across breeds requires accurate estimation of QTL effects. We show that accuracy of across-breed genomic predictions was higher with BayesR than with GBLUP and that BayesR mapped QTL more precisely. Further improvements of across-breed accuracy of genomic predictions and QTL mapping could be achieved by increasing the size of the reference population, including more breeds, and possibly by exploiting pleiotropic effects to improve mapping efficiency for QTL with small effects.

**Electronic supplementary material:**

The online version of this article (doi:10.1186/s12711-014-0074-4) contains supplementary material, which is available to authorized users.

## Background

The accuracies of genomic predictions are often reported to decrease with increasing genetic distance (or meiosis) from the reference population. For example, Habier *et al*. [1] showed that, in the German Holstein breed, accuracies of genomic predictions of animals that were distantly-related to the reference population declined. Saatchi *et al*. [2] reported a decline in accuracy of genomic predictions that were derived from a US Hereford population when they were tested in Canadian, Uruguayan or Argentinean Hereford populations. These results suggest that the linkage disequilibrium (LD) between markers and quantitative trait loci (QTL) was different in the validation population compared to the reference or training population. This occurs because LD within a group of related animals may be lost due to recombination in less closely-related animals. Several authors also reported that the accuracy of genomic predictions was poor for a breed not included in the reference (i.e. across-breed genomic predictions) [3,4]. Across-breed prediction is particularly challenging because, in addition to the possible occurrence of inconsistent LD between markers and QTL [5,6], QTL may be breed-specific, which places an upper limit to the accuracy that can be reached in another breed.

This problem of poor prediction for animals not closely-related to the reference population is exacerbated when BLUP (best linear unbiased prediction) is used to derive prediction equations. BLUP (or the mathematical equivalent genomic BLUP, GBLUP) is widely used for genomic prediction because of its computational efficiency and because it performs almost as well as nonlinear methods for within-breed prediction [7,8]. GBLUP assumes that the effects of all markers are drawn from the same normal distribution, which implies that all markers are assumed to have very small effects. In spite of this unrealistic assumption, GBLUP can capture the effects of QTL, even if the effects are moderate to large, by using a linear combination of markers. Since LD can extend over long genomic distances, this linear combination can include markers that are a long distance away from the QTL. For example, long-range LD probably explains why predictions based on 50 K single nucleotide polymorphism (SNP) markers have similar accuracies as predictions based on higher density chips (800 K) for within-breed prediction of Holstein cattle [9,10]. Thus, closely-related animals inherit similar long chromosomal segments to those of the animals in the reference population and hence the same linear combination of markers will predict the effect of QTL. However, if recombination breaks up these long chromosomal segments, the predictive power of the linear combination of markers will decrease [1]. In contrast, non-linear methods, such as BayesB [11], allow the effects of some markers to be large, while many markers have zero (or near-zero) effect. This allows the prediction equation to be driven by markers that are close to the QTL and in strong LD with it. The LD between such markers and their associated QTL is broken down less quickly because the recombination distance between them is small. Although using non-linear alternatives (e.g. BayesA, BayesB, BayesR) is not always superior to GBLUP for within-breed prediction, nonlinear methods are expected to improve the persistency of the accuracy of genomic predictions over future generations [1,10].

Within a single breed, a marker may be in strong LD with a QTL in spite of being some distance away. Therefore, to find markers close to and in LD with QTL in all breeds, using a reference population that includes more than one breed can be advantageous. Combining animals from multiple breeds in a reference dataset will reduce the long-range LD that is present within a breed but may not necessarily increase the accuracy of predictions, particularly if predictions are evaluated in direct offspring of the reference population. Thus, it is not surprising that, in the literature, the reported benefits of using a multi-breed reference dataset are mixed. However, some improvements have been observed for breeds with small (within-breed) reference populations and, in general, results have been more promising for beef cattle than for dairy cattle [4,12-16]. In some cases, the failure of prediction equations to benefit from a multi-breed reference population could be due to the use of medium (50 K) density SNP chips, which are unlikely to have consistent across-breed LD [6].

In this paper, we show that using a large reference population from two breeds, combined with high-density SNP genotypes and a nonlinear method for the analysis (BayesR) increases the accuracy of genomic predictions in a breed that is not included in the reference population. To create a large reference population, we expanded the current Australian reference population of progeny-tested bulls by including cows e.g. [16,17]. Our dataset of bulls and cows is similar to that recently used by Raven *et al*. [18] for a genome-wide association study. Here, in contrast to [18], we fitted all SNPs simultaneously and extended the BayesR methodology from Erbe *et al*. [10] to include cow records and estimate fixed effects. The use of cows requires making changes in the evaluation procedure because cows and bulls have different degrees of uncertainty in their measurement, i.e. there is heterogeneous error variance. In addition, if nonlinear methods identify markers that are close to QTL, they should be able to map the QTL with greater precision than alternative methods such as GBLUP. We assessed the ability of BayesR with the multi-breed reference to fine-map QTL by mapping known loci such as *DGAT1* [19] and identify new genes that affect dairy traits by combining the BayesR results with differential gene expression of the mammary gland compared to that of 17 other tissue types.

## Methods

### Data

#### Genotypes

**Figure 1:**
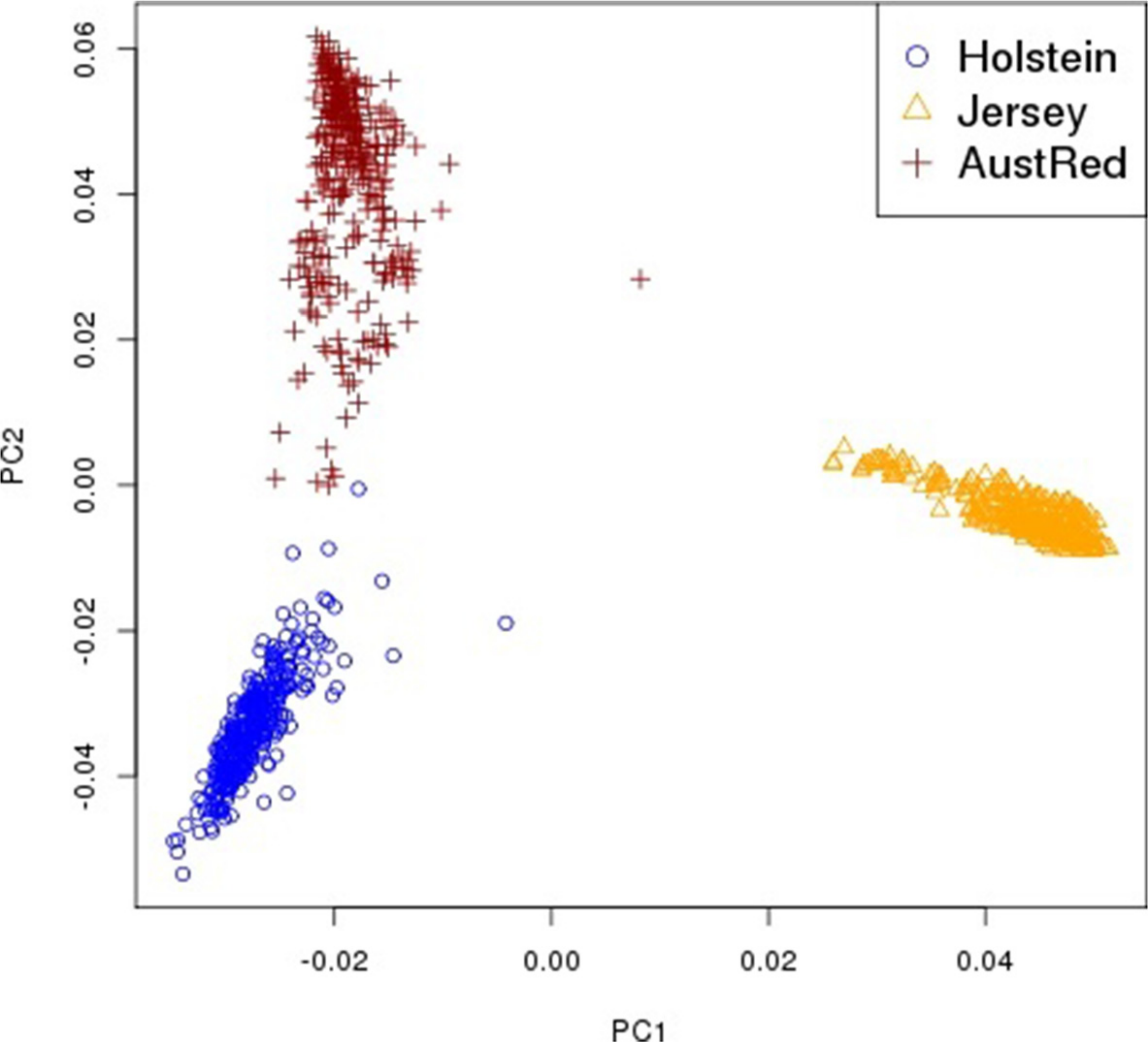
**Relationships between Holstein, Jersey and Australian Red dairy cattle.** Shown are principal components 1 and 2 for the genomic relationship matrix [24] constructed from a random sample of Holstein (n =334) and Jersey (n =326) animals with the genotyped Australian Red (n =313) animals. Principle components were obtained using the eigen() function in R [50].

Illumina Bovine HD genotypes (777 K SNPs) were available for 1620 Holstein bulls and cows, 125 Jersey bulls, and 114 Australian Red bulls. After quality control, carried out as in Erbe *et al*. [10], and removal of non-polymorphic SNPs, 632 002 SNPs remained. A total of 10 311 Holstein, 4738 Jersey and 249 Australian Red bulls and cows were genotyped with the Illumina Bovine SNP array (54 K SNPs) and passed parentage verification. After quality control, 43 425 SNPs remained. All animals had genotypes imputed to the higher density SNP panel using Beagle 3 [20]. The Australian Red animals were used only to evaluate the prediction equations derived from reference populations of Holstein animals, Jersey animals or Holstein plus Jersey animals. Australian Red cattle have a large component of Scandinavian Red ancestry and are genetically distinct from Holstein and Jersey cattle (Figure 1). The average LD between markers for Australian Red cattle is lower than that of either Holstein or Jersey cattle (see Additional file 1: Figure S1).

#### Phenotypes

Phenotypes for the genotyped animals (trait deviations for cows and daughter trait deviations for bulls) were from the April 2013 genetic evaluations from the Australian Dairy Herd Improvement Scheme (ADHIS) for fat yield (FY), milk yield (MY), protein yield (PY), fertility (FERT), stature (STAT) and survival (SURV). Trait deviations were corrected for herd year season effects, permanent environmental effects, and heterosis. Milk composition traits, i.e. percentage of fat and protein in milk (F% and P%), were calculated using a linear approximation of the milk production and milk solid yield traits. For example, F% was calculated as:

i $$ F\%=\frac{F{Y}_P}{M{Y}_P}\left[\frac{FY}{F{Y}_P}-\frac{MY}{M{Y}_P}\right], $$

where *FY*_*P*_ is the (within-breed) average fat yield and *MY*_*P*_ is the (within-breed) average milk yield. P% was calculated using the same methodology. Values for *FY*_*P*_ (kg/lactation in Holstein = 284; Jersey = 522; Australian Red = 256), *PY*_*P*_ (kg/lactation in Holstein = 243; Jersey = 193; Australian Red = 216) and *MY*_*P*_ (L/lactation in Holstein = 7417; Jersey = 5273; Australian Red = 6254) were from the 2012 *ADHIS* annual report [21].

### Reference and validation datasets

The Holstein and Jersey phenotypes were split into reference and validation datasets for each trait. The reference datasets consisted of six different combinations of up to 11 527 Holstein and 4687 Jersey animals. The six reference sets were: (1) Holstein bulls, (2) Jersey bulls, (3) Holstein and Jersey bulls, (4) Holstein bulls and cows, (5) Jersey bulls and cows or (6) all reference animals (Holstein and Jersey bulls and cows).

The four validation datasets consisted of a minimum of: (1) 251 Holstein bulls, (2) 81 Jersey bulls, (3) 247 Australian Red cows or (4) 114 Australian Red bulls. Validation animals for the Holstein and Jersey breeds were selected on the basis of birth year and cows that were progeny of bulls in the validation set were removed from the reference set. All bulls had more than 20 effective daughter records. The number of animals in the reference and validation populations for each breed and each trait are in Table 1.

**Table 1:** **Number of phenotypic records for each trait in the reference and validation sets**

				**Total**	**Reference**		**Validation**	
**Breed**	**Trait**	**h** ^2^	**r**	**records**	**Bulls**	**Cows**	**YOB**	**Bulls**
Holstein	FY, F%, MY, PY, P%	0.33	0.56	11 789	3049	8478	2005	262
Holstein	STAT	0.45	1	4481	1484	2746	2003	251
Holstein	FERT	0.03	0.05	11 040	2806	7838	2004	396
Holstein	SURV	0.025	0.035	10 999	2810	7825	2004	364
Jersey	FY, F%, MY, PY, P%	0.33	0.56	4793	770	3917	2005	105
Jersey	STAT	0.45	1	2552	300	2167	2001	85
Jersey	FERT	0.03	0.05	4628	716	3830	2005	81
Jersey	SURV	0.025	0.035	4592	697	3791	2004	103
Australian Red	FY, F%, MY, PY, P%	*Validation animals only, 247 cow and 114 bull records.*						

FY = fat yield (kg/lactation), MY = milk yield (L/lactation), PY = protein yield (kg/lactation), F% = fat percentage (%) and P% = protein percentage in milk (%); STAT = stature; FERT = fertility (calving interval, days); and SURV = daughter survival (annual probability); *h* ^2^ = trait heritability, *r* = trait repeatability, YOB = oldest year of birth. *h* ^2^ and *r* were assumed for each trait when calculating the weights in the reference population.

### Model fitted to the reference data

Genomic predictions for each trait were estimated for the validation datasets using only animals in the prescribed reference dataset. Two procedures were used to estimate marker effects, either GBLUP or BayesR. The model that was fitted to the reference dataset in both cases included fixed effects (overall mean, breed and sex nested within breed, when appropriate), SNP effects and polygenic effects. The model was:

ii $$ \mathbf{y}=\mathbf{X}\mathbf{b}+\mathbf{Z}\mathbf{a}+\mathbf{W}\mathbf{v}+\mathbf{e}, $$

where:

**y** = vector of *n* trait or daughter deviations (phenotypes) for cows or bulls,

**b** = vector of *p* fixed effect solutions,

**a** = vector of *q* polygenic breeding values, distributed *N*(0, **A***σ*^2^_*a*_),

**v** = vector of *m* SNP effects,

**e** = vector of *n* residual errors, distributed *N*(0, **E***σ*^2^_*e*_),

**X** = design matrix allocating phenotypes to fixed effects (**X** = *n* by *p* matrix),

**Z** = design matrix allocating phenotypes to polygenic breeding values (***Z*** = *n* by *q* matrix),

**W** = design matrix of SNP marker genotypes (**W** = *n* by *m* matrix),

**A** = numerator relationship matrix,

*σ*^2^_*a*_ = additive genetic variance not explained by the SNPs,

*σ*^2^_*e*_ = error variance.

#### Constructing the matrix of weights for errors (E)

The analysis aimed to account for the uncertainty in phenotypic records, particularly between bulls and cows but also for bulls with few or many daughters. This uncertainty affects the error variance associated with each record, that is *e ~ N*(0,**E***σ*^2^_*e*_)*,* where **E** is a diagonal matrix constructed as *diag(1/w*_*i*_*)*, where *w*_*i*_ is the weighting coefficient for each animal. Weights were scaled such that the error variance for animals with one observation of their own phenotype is *σ*^2^_*e*_. The calculation of the weighting coefficient differs between cows (which have their own records) and bulls (for which phenotypes are daughter deviations) and was done following Garrick *et al*. [22] i.e.:

iii $$ {w}_i(bulls)=\frac{d\left(1-{h}^2\right)}{4-{h}^2}, $$

iv $$ {w}_i(cows)=\frac{r\left(1-{h}^2\right)}{1+\left(r-1\right)t-r{h}^2}, $$

where *h*^2^ is the heritability of a single record of the trait, *d* is the effective number of daughters, *r* is the number of records per cow and *t* is the repeatability of the trait. All variables (*h*^2^, *d*, *r* and *t*) were obtained from ADHIS, and the heritabilities and repeatabilities for each trait are in Table 1.

#### GBLUP

The GBLUP method was implemented using restricted maximum likelihood in ASReml [23]. GBLUP assumes that all marker effects are drawn from the same distribution [i.e. $\mathbf{v}\sim N\left(0,\mathbf{I}{\sigma}_v^2\right)$] and a model equivalent to Equation (ii) was fitted in which **Wv** = **Qg**, where **Q** is a (*n* x *n*) design matrix allocating phenotypes to animals and $\mathbf{g}\sim N\left(0,\mathbf{G}{\sigma}_g^2\right)$]. **G** was calculated according to Yang *et al.* [24] and ${\sigma}_g^2$ is the genetic variance explained by all SNPs and was estimated from the data. Solutions for fixed effects ($\widehat{\mathbf{b}}$), polygenic breeding values (**â**) and genomic estimated breeding values (**ĝ**) for the model **y** = **Xb** + **Za** + **Qg** + **e** are the same as for the mixed model equations following [25]:

v $$ \left[\begin{array}{ccc}\hfill \mathbf{X}\hbox{'}{\mathbf{R}}^{-1}\mathbf{X}\hfill & \hfill \mathbf{X}\hbox{'}{\mathbf{R}}^{-1}\mathbf{Z}\hfill & \hfill \mathbf{X}\hbox{'}{\mathbf{R}}^{-1}\mathbf{Q}\hfill \\ {}\hfill \mathbf{Z}\hbox{'}{\mathbf{R}}^{-1}\mathbf{X}\hfill & \hfill \mathbf{Z}\hbox{'}{\mathbf{R}}^{-1}\mathbf{Z}+{\mathbf{A}}^{-1}{\sigma}_a^{-2}\hfill & \hfill \mathbf{Z}\hbox{'}{\mathbf{R}}^{-1}\mathbf{Q}\hfill \\ {}\hfill \mathbf{Q}\hbox{'}{\mathbf{R}}^{-1}\mathbf{X}\hfill & \hfill \mathbf{Q}\hbox{'}{\mathbf{R}}^{-1}\mathbf{Z}\hfill & \hfill \mathbf{Q}\hbox{'}{\mathbf{R}}^{-1}\mathbf{Q}+{\mathbf{G}}^{-1}{\sigma}_g^{-2}\hfill \end{array}\right]\left[\begin{array}{c}\hfill \widehat{\mathbf{b}}\hfill \\ {}\hfill \widehat{\mathbf{a}}\hfill \\ {}\hfill \widehat{\mathbf{g}}\hfill \end{array}\right]=\left[\begin{array}{c}\hfill \mathbf{X}\hbox{'}{\mathbf{R}}^{-1}\mathbf{y}\hfill \\ {}\hfill \mathbf{Z}\hbox{'}{\mathbf{R}}^{-1}\mathbf{y}\hfill \\ {}\hfill \mathbf{Q}\hbox{'}{\mathbf{R}}^{-1}\mathbf{y}\hfill \end{array}\right], $$

where **R**^−1^ = **E**^−1^*σ*^−2^_*e*_, and all other terms are as described above. Following [25], the solutions are:

vi $$ \widehat{\mathbf{b}}={\left[{\mathbf{X}}^{\hbox{'}}{\mathbf{R}}^{-1}\mathbf{X}\right]}^{-1}\mathbf{X}\hbox{'}{\mathbf{R}}^{-1}\left(\mathbf{y}-\mathbf{Z}\widehat{\mathbf{a}}-\mathbf{Q}\widehat{\mathbf{g}}\right), $$

vii $$ \widehat{\mathbf{a}}={\left[{\mathbf{Z}}^{\hbox{'}}{\mathbf{R}}^{-1}\mathbf{Z}+{\mathbf{A}}^{-1}{\sigma}_a^{-2}\right]}^{-1}\mathbf{Z}\hbox{'}{\mathbf{R}}^{-1}\left(\mathbf{y}-\mathbf{X}\widehat{\mathbf{b}}-\mathbf{Q}\widehat{\mathbf{g}}\right), $$

viii $$ \widehat{\mathbf{g}}={\left[{\mathbf{Q}}^{\hbox{'}}{\mathbf{R}}^{-1}\mathbf{Q}+{\mathbf{G}}^{-1}{\sigma}_g^{-2}\right]}^{-1}\mathbf{Q}\hbox{'}{\mathbf{R}}^{-1}\left(\mathbf{y}-\mathbf{X}\widehat{\mathbf{b}}-\mathbf{Z}\widehat{\mathbf{a}}\right). $$

In this study, solutions for $\hat{\mathbf{g}}$ were obtained with ASReml [23] and then back-solved to estimate SNP effects, i.e. to obtain solutions for $\hat{\mathbf{v}}$, where back-solving was as described by Yang *et al*. [26].

#### BayesR

This paper extends BayesR, following Meuwissen *et al.* [11], Meuwissen and Goddard [27] and Erbe *et al.* [10], with modifications to account for the heterogeneous error variance in the phenotypes and to estimate the fixed effects in the model. BayesR [10] assumes that the distribution of SNP effects is a mixture of normal distributions, with the k^th^ component comprising a proportion pr_k_ of the mixture and having variance *σ*^*2*^_*k*_. Similar to the construction of the **G** matrix [24], SNP alleles in **W** were standardised prior to analysis in BayesR to have a unit variance (i.e. $\left[{w}_{i,j}-2 freq\left({w}_j\right)\right]/\sqrt{2 freq\left({w}_j\right)\left(1- freq\left({w}_j\right)\right)}$, where *w*_*i,j*_ is the genotype of SNP *j* for animal *i*, and freq(*w*_*j*_) is the allele frequency of *j*). Note that in the following, the current estimates of the parameters in the Gibbs sampler used in the analysis (e.g. $\tilde{\mathbf{b}}$) are distinguished from the final estimates (e.g. $\widehat{\mathbf{b}}$) using superscript notation. The model is implemented using the following steps:

1. The error variance was sampled from a scaled inverse chi-squared distribution with mean equal to **ẽE**^− 1^**ẽ** and *n – 2* degrees of freedom where $\tilde{\mathbf{e}}=\mathbf{y}-\mathbf{X}\tilde{\mathbf{b}}-\mathbf{Z}\tilde{\mathbf{a}}-\mathbf{W}\tilde{\mathbf{v}}$, and $\tilde{\mathbf{b}}$, **ã** and **ṽ** are the current values of those terms in the model.
2. The fixed effects were sampled from a normal distribution with a mean given by [**X**^'^**R**^− 1^**X**]^− 1^**X**^'^**R**^− 1^y*, following Equation (vi) where y* is the phenotype corrected for the current estimates of all other terms and fixed effects in the model, with variance [**X**^'^**R**^− 1^**X**]^− 1^.
3. The polygenic effect was sampled from a normal distribution, with mean for animal *i* equal to ${\left[{{\mathbf{Z}}_i}^{\hbox{'}}{\mathbf{R}}_{ii}^{-1}{\mathbf{Z}}_{\mathrm{i}}+{\boldsymbol{A}}_{ii}^{-1}{\upsigma}_a^{-2}\right]}^{-1}{\mathbf{Z}}_i^{\hbox{'}}$, following Equation (vii), where **Z**_*i*_ is the row corresponding to animal *i* in **Z** and **A**_*ii*_^−1^ and **R**_*ii*_^−1^ are the i^th^ diagonal elements of **A**^−1^ and **R**^−1^, respectively. The variance of the sampling distribution for the polygenic effect for animal *i* was ${\left[{{\mathbf{Z}}_i}^{\hbox{'}}{\mathbf{R}}_{ii}^{-1}{\mathbf{Z}}_i+{\mathbf{A}}_{ii}^{-1}{\upsigma}_a^{-2}\right]}^{-1}$. More details on the estimation of the polygenic effects are in the appendix of [28].
4. The polygenic variance was sampled from a scaled inverse chi-squared distribution with mean **ãA**^− 1^**ã** and *n – 2* degrees of freedom.
5. The effect of SNP *j* was sampled by first sampling a component of the mixture and then drawing *ṽ*_*j*_ from that distribution. A residual model (i.e. ${\mathbf{y}}_j^{*}={\mathbf{W}}_{.j}{v}_j+{\mathbf{e}}_{\boldsymbol{j}}$, where **y**_j_^*^ is the phenotype corrected for all other effects, excluding the current marker *j*, **W**_*.j*_ is column *j* from the genotype matrix **W**, *v*_*j*_ is the allelic substitution effect of marker *j*, and **e**_***j***_ is the error) was used to determine the full conditional posterior probability for each distribution *k* as:
  $$ L\left({v}_{j,k}\Big|{\sigma}_k^2\right)=-0.5\left[ \ln \left(1+{\mathbf{W}}_{.j}\hbox{'}{\mathbf{R}}^{-1}{\mathbf{W}}_{.j}{\sigma}_k^2\right)+{\mathbf{y}}_j^{*}\hbox{'}{\mathbf{R}}^{-1}{\mathbf{y}}_j^{*}-{\mathbf{y}}_j^{*}\hbox{'}{\mathbf{R}}^{-1}{\mathbf{W}}_{.j}{v}_{j,k}\right]+ \ln \left(p{r}_k\right), $$
  where *pr*_*k*_ is the current estimate for the proportion of markers from distribution *k*, *v*_*j*,*k*_ is an estimate of the effect for marker *j* when sampled from distribution *k*, and ${v}_{j,k}={\left[{\mathbf{W}}_{.j}\hbox{'}{\mathbf{R}}^{-1}{\mathbf{W}}_{.j}+{\sigma}_k^{-2}\right]}^{-1}{\mathbf{W}}_{.j}\hbox{'}{\mathbf{R}}^{-1}{\mathbf{y}}^{*}$ (following Equation (vi))*.* The full conditional posterior probability that marker *j* is from distribution *k* was calculated as:
  $$ {\left\{{\displaystyle \sum_{l=1,4}} \exp \left[L\left({v}_{j,l}\Big|{\sigma}_l^2\right)-L\left({v}_{j,k}\Big|{\sigma}_k^2\right)\right]\right\}}^{-1}. $$
  More details for the derivation of the full conditional likelihood function are in Additional file 2 (see Additional file 2). Once the component of the mixture distribution was determined, allele effects were sampled from a normal distribution, using the residual model with a mean *v*_*j*,*k*_ and variance ${\left[{\mathbf{W}}_{.j}\hbox{'}{\mathbf{R}}^{-1}{\mathbf{W}}_{.j}+{\sigma}_k^2\right]}^{-1}$.
6. The prior *pr*_*k*_ was updated by sampling from a Dirichlet distribution given by *pr*_*k*_ ~ Dir(*α*_*k*_ + *β*_*k*_), where *α*_*k*_ is the prior counts for markers from distribution *k* and *β*_*k*_ is the current number of markers with effects sampled from distribution *k*. The prior assumed one marker from each distribution (i.e. *α*_*k*_ =1).

BayesR was implemented with a multi-threaded C++ program to improve computing performance. Based on Erbe *et al*. [10], we defined four possible distributions for *σ*^2^_*v,k*_ with variance equal to 0, 0.0001*σ*^2^_a2_, 0.001*σ*^2^_a2_, and 0.01*σ*^2^_a2_, where *σ*^2^_*a2*_ is the additive genetic variance explained by the pedigree, which was determined prior to the analysis by fitting **y** = **Xb** + **Za** + **e** (following Equation (ii)) in ASReml [23]. The Gibbs sampler used at least 30 000 iterations, with 20 000 iterations discarded as burn-in, and each analysis had five replicate Gibbs sampling chains. Final parameter estimates were the means of the sampled effects in the post burn-in iterations, which were obtained separately for each chain.

### Assessment of the accuracy and bias of predictors

The model that was fitted to the reference datasets always included the estimate of marker effects ($\widehat{\mathbf{v}}$) and a polygenic (**â**) term. Thus, predictions for the Holstein and Jersey validation datasets considered only predictions based on genotype (${\widehat{\mathbf{y}}}_{\mathbf{v}}=\mathbf{W}\widehat{\mathbf{v}}$) or prediction of the total genetic merit of the animal ($\widehat{\mathbf{y}}=\widehat{\mathbf{a}}+\mathbf{W}\widehat{\mathbf{v}}$). A proxy for the accuracy of prediction was assessed for these two quantities as the correlation between the prediction and the phenotype [i.e. $r\left(\mathbf{y},\widehat{{\mathbf{y}}_{\mathbf{v}}}\right)$ or *r*(**y**, **ŷ**)] and the bias in the prediction was assessed as the regression coefficient of phenotype on the prediction [i.e. $b\left(\mathbf{y},\widehat{{\mathbf{y}}_{\mathbf{v}}}\right)$ or *b*(**y**, **ŷ**)]. For BayesR, the accuracy was the average correlation across the five replicate chains.

Accuracies were calculated for many combinations of dataset (bulls or bulls and cows), for each method (GBLUP or BayesR), breed (single or both breeds), and with or without inclusion of the polygenic term in the prediction. Therefore, to summarize the effects of all these factors on accuracy, the accuracies (r) were analysed using the following linear model r_m,n,o,p_ = dataset_m_ + dataset_m_.method_n_ + dataset_m_.breed_o_ + dataset_m_.polygenic_p_ + e_m,n,o,p_. We did not use this model to test significance of each factor because the accuracies were not independent. Rather, we used the model to estimate the effect of each factor and reported these estimates. Data on bias were analysed in the same way.

### Derterminating the precision of QTL mapping

To map QTL, GEBV in sliding windows of 250 kb (i.e. ‘local’ GEBV) [29] were calculated for each animal from the multi-breed bull and cow reference dataset for the milk production traits (FY, MY, PY, F%, P%). Local GEBV were calculated as ${\mathbf{W}}_{{\boldsymbol{j}}_1:{\boldsymbol{j}}_2}{\widehat{\mathbf{v}}}_{{\boldsymbol{j}}_1:{\boldsymbol{j}}_2}$, where ${\mathbf{W}}_{{\boldsymbol{j}}_1:{\boldsymbol{j}}_2}$ and ${\widehat{\mathbf{v}}}_{{\boldsymbol{j}}_1:{\boldsymbol{j}}_2}$ includes all SNP markers within a 250 kb region of the genome. Adjacent 250 kb windows were separated by 50 kb. The variance of the local GEBV was determined for each breed, trait and window. If the variance of a window was greater than 50 times that of an average window, the window was defined as containing a QTL. Windows that contained QTL were examined for possible candidate loci based on QTL reported in the literature and for genes that were over- or under-expressed in the mammary gland (P < 1 × 10^−5^) compared to 17 other tissue types [30]. To obtain the latter, RNA was extracted from 18 tissues (including mammary gland) in triplicate from a single lactating Holstein cow at one time point. RNA was sequenced on the Illumina HiSeq2000 platform using 100 base paired end reads. After quality control and filtering, approximately 4 × 10^7^ to 1 × 10^8^ reads per tissue were aligned to the Ensembl annotation of the UMD3.1 bovine genome assembly using Tophat2 [31]. A matrix of gene counts by tissue was constructed with HTSeq [32] and the bioconductor ‘edgeR’ package [33] was used to perform tissue-specific expression analysis where the intercept was the mean gene expression across all tissues.

QTL often affected more than one milk production trait. We summarised the pleiotropic pattern of the effects on milk production traits of windows that were identified to contain QTL as follows. The correlation between local GEBV [34] for all pairwise combinations of traits were calculated for windows for which the local GEBV variance for each trait was greater than 3 times that of an average window. Windows with mid-points separated by less than 0.5 Mbp and with similar patterns of effects were assumed to be part of the same QTL and combined into a single region. QTL were allocated to one of nine possible groups, first according to the QTL’s largest effect on a yield trait (FY, MY, PY) and then by the QTL’s pattern of pleiotropic effects on the remaining two yield traits (defined by either a negative (−) or positive (+) correlation, or with no (*n*) effect. For example, a 'FY-' QTL had its largest effect on FY and a negative correlation with (either one or both) of MY and PY. Similarly, a 'MY*n*' QTL had its largest effect on MY with no notable effect on either FY or PY. QTL regions affecting only P% were grouped with the MY*n* QTL as a change in milk composition was assumed to be a sensitive measure of increased milk volume (i.e. an increase in milk volume with no change in milk solids would result in a decreased P%. Hence P% was considered to be potentially more sensitive to changes in MY than to changes in milk volume than L of milk per lactation).

## Results

### Variance components from GBLUP and BayesR

The proportions of phenotypic variance captured by polygenic effects, SNPs and residuals for each method were investigated to assess differences between the GBLUP and BayesR. The proportion of phenotypic variance captured by genetic terms (i.e. SNP + polygenic) in GBLUP and BayesR differed by less than 5% for most traits (Table 2). The notable exception was F% (Table 2), for which the BayesR estimate of the genetic variance (SNP + polygenic) was 20 to 30% smaller than the GBLUP estimate.

**Table 2:** **Variance components from GBLUP and BayesR for the combined (bull and cow) reference sets**

**Breed**	**Trait**	**Pedigree estimate**		**GBLUP estimate**				**BayesR estimate**			
		***σ*** ^2^_***P***_	***h*** ^2^_a2_	***σ*** ^2^_***P***_	***h*** ^2^_***v***_	***h*** ^2^_***a***_	***h*** ^2^_***v***_ ***/(*** ***h*** ^2^_***v***_ **+** ***h*** ^2^_***a***_ **)**	***σ*** ^2^_***P***_	***h*** ^2^_***v***_	***h*** ^2^_***a***_	***h*** ^2^_***v***_ ***/(*** ***h*** ^2^_***v***_ **+** ***h*** ^2^_***a***_ ***)***
Holstein	FY	437.43	0.428	420.05	0.273	0.122	0.692	416.23	0.242	0.146	0.623
Holstein	MY	341880	0.533	321395	0.361	0.134	0.729	307529	0.312	0.176	0.639
Holstein	PY	272.29	0.469	262.13	0.273	0.142	0.658	263.62	0.255	0.166	0.606
Holstein	F%	0.0839	0.728	0.0776	0.628	0.098	0.865	0.0613	0.473	0.181	0.723
Holstein	P%	0.0148	0.864	0.0132	0.643	0.136	0.825	0.0137	0.597	0.188	0.760
Holstein	FERT	3335	0.014	3260	0.014	0.000	1.000	3269	0.014	0.001	0.948
Holstein	SURV	0.0698	0.025	0.0690	0.019	0.003	0.849	0.0594	0.023	0.009	0.708
Holstein	STAT	1.50	0.244	1.42	0.225	0.007	0.969	1.45	0.225	0.020	0.918
Jersey	FY	359.48	0.534	366.59	0.298	0.175	0.630	358.44	0.276	0.150	0.648
Jersey	MY	226780	0.606	226188	0.402	0.186	0.684	213026	0.371	0.163	0.695
Jersey	PY	219.18	0.539	221.68	0.292	0.182	0.616	220.37	0.281	0.167	0.628
Jersey	F%^*^	0.1013	0.992	0.1051	0.648	0.335	0.660	0.0735	0.566	0.283	0.666
Jersey	P%	0.0254	0.863	0.0243	0.695	0.188	0.787	0.0219	0.611	0.183	0.770
Jersey	FERT	3975	0.004	3928	0.005	0.000	1.000	3890	0.005	0.001	0.891
Jersey	SURV	0.0456	0.051	0.0455	0.029	0.020	0.587	0.0453	0.028	0.020	0.578
Jersey	STAT	0.76	0.405	0.76	0.297	0.093	0.762	0.75	0.294	0.075	0.796
Hol/Jer	FY	413.96	0.454	404.82	0.276	0.137	0.668	405.77	0.248	0.159	0.610
Hol/Jer	MY	307160	0.556	293243	0.373	0.148	0.715	288223	0.340	0.180	0.654
Hol/Jer	PY	256.40	0.487	250.61	0.276	0.154	0.642	256.90	0.272	0.169	0.617
Hol/Jer	F%	0.0895	0.795	0.0866	0.633	0.143	0.816	0.0688	0.503	0.201	0.714
Hol/Jer	P%	0.0179	0.844	0.0164	0.636	0.159	0.800	0.0178	0.621	0.169	0.786
Hol/Jer	FERT	3530	0.011	3465	0.012	0.000	1.000	3452	0.012	0.000	0.963
Hol/Jer	SURV	0.0627	0.029	0.0622	0.021	0.006	0.761	0.0626	0.021	0.009	0.710
Hol/Jer	STAT	1.20	0.312	1.15	0.267	0.026	0.912	1.19	0.268	0.038	0.877

FY = fat yield (kg/lactation), MY = milk yield (L/lactation), PY = protein yield (kg/lactation), F% = fat percentage (%) and P% = protein percentage in milk (%); STAT = stature; FERT = fertility (calving interval, days); and SURV = daughter survival (annual probability); *σ* ^2^_*P*_ = phenotypic variance, and ratios of *h* ^2^_*v*_ = *σ* ^2^_*v*_/*σ* ^2^_*P*_ (where *σ* ^*2*^_*v*_ = variance explained by SNPs) and *h* ^2^_*a*_ = *σ* ^2^_*a*_/*σ* ^2^_*P*_ (where *σ* ^*2*^_*a*_ = additive genetic variance) or *h* ^2^_*a*2_ = *σ* ^2^_*a*2_/*σ* ^2^_*P*_ (where *σ* ^2^_*a2*_ = additive genetic variance, when SNPs are not included in the model); ^*^due to singularities, variance components for F% in Jersey using the pedigree were estimated using an unweighted analysis.

The total genetic variance accounted for less than 5% of the phenotypic variance for FERT and SURV; 20 to 60% of the phenotypic variance for FY, MY, PY and STAT; and more than 70% of the phenotypic variance for F% and P% (Table 2). The variance captured by SNPs was equal to about 70% of the genetic variance for production traits (FY, MY, PY, F%, P%) and about 90% for STAT and FERT. For SURV, the proportion of genetic variance captured by SNPs in Jersey cattle was low (less than 60%) compared to the estimate in either the Holstein or the multi-breed reference datasets (about 75%). BayesR and GBLUP resulted in very similar estimates of the variance captured by SNPs, relative to the total genetic variance, in Jersey cattle for most traits. However, SNPs in the Holstein and the multi-breed Holstein/Jersey reference datasets were estimated to capture 5 to 10% less of the total genetic variance with BayesR than with GBLUP.

The average number of SNPs in each distribution for BayesR indicates that most SNPs (>99%) had no effect on the traits (Table 3). More than 10 SNPs were estimated to come from the distribution with the largest variance (i.e. 0.01*σ*^2^_*a2*_) for P% and F% in the Jersey and the multi-breed Holstein/Jersey reference datasets, and for P%, STAT and FERT in the Holstein dataset. Between 5 and 10 SNPs were estimated from the distribution with the largest variance for FY, F% in Holstein cattle; for FY, MY, PY, STAT and FERT in Jersey cattle and for FY, MY, STAT and FERT in the multi-breed Holstein/Jersey reference dataset. SURV had the lowest number of SNPs estimated from the distribution with the largest variance for all traits for both the Holstein and Jersey datasets.

**Table 3:** **Average number of SNPs estimated to be in each distribution by BayesR** ^1^

**Breed**	**Trait**	**0.0001** ***σ*** ^2^_a2_	**0.001** ***σ*** ^2^_a2_	**0.01** ***σ*** ^2^_a2_
Holstein	FY	3968.0	53.4	7.4
Holstein	MY	3834.4	78.0	5.8
Holstein	PY	4352.4	39.6	4.6
Holstein	F%	2451.8	70.2	9.2
Holstein	P%	2376.8	175.2	13.0
Holstein	STAT	5685.0	241.0	10.4
Holstein	FERT	5874.2	163.4	13.4
Holstein	SURV	2731.8	39.6	4.6
Jersey	FY	3897.2	48.8	7.6
Jersey	MY	2960.0	68.2	6.4
Jersey	PY	3469.0	72.6	6.0
Jersey	F%	2562.8	94.0	23.4
Jersey	P%	3318.4	152.0	40.2
Jersey	STAT	2472.6	295.6	7.0
Jersey	FERT	1303.0	125.0	9.8
Jersey	SURV	1935.0	116.8	3.0
Hol/Jer	FY	4388.6	23.2	6.2
Hol/Jer	MY	4155.4	54.2	6.2
Hol/Jer	PY	4583.2	36.6	4.6
Hol/Jer	F%	3145.2	55.2	11.6
Hol/Jer	P%	3591.2	178.2	19.6
Hol/Jer	STAT	5773.0	225.8	9.2
Hol/Jer	FERT	5575.0	142.0	9.4
Hol/Jer	SURV	2781.2	29.0	4.2

FY = fat yield (kg/lactation), MY = milk yield (L/lactation), PY = protein yield (kg/lactation), F% = fat percentage (%) and P% = protein percentage in milk (%); STAT = stature; FERT = fertility (calving interval, days); and SURV = daughter survival (annual probability); the number of SNPs in the zero distribution (632 003 minus the sum of the SNPs from the three other distributions) is not shown; ^1^where *σ* ^*2*^_a2_ is the additive genetic variance estimated with pedigree (only).

### Assessment of the accuracy and bias of predictions

Averaged across the five milk production traits, the accuracy of Holstein GBLUP predictions using bulls only in the reference dataset was equal to 0.61, compared with 0.52 if only pedigree information (no SNP effects) was used (see Additional file 3: Table S1). Increasing the size of the reference dataset by including cow records had the largest and most consistent effect on improving the accuracy of genomic predictions for milk production traits (FY, MY, PY, F%, P%) and STAT. The accuracy increased by an average of 5.4% in the Holstein and 4.2% in the Jersey breed when cows were added to the reference datasets for these traits. However, there was little or no benefit of adding cows to the reference dataset for FERT in the Holstein breed and for SURV in each breed. The effect of adding Jersey animals into the combined (bull and cow) Holstein reference dataset had little effect on the accuracies for milk production traits in the Holstein breed, but there was a small average increase in accuracy of 1% for milk production traits in the Jersey breed. Genomic predictions for all Jersey and Holstein validation populations are in Additional file 3 (see Additional file 3: Table S2 and Table S3). Table S1 (see Additional file 3: Table S1) summarises the effects of the reference dataset, the method of prediction and the addition of the polygenic term on the accuracy and bias of predictions.

Accuracies obtained with the BayesR method were generally equal to or higher than those with GBLUP (see Additional file 3: Table S1). The average increase in accuracy using BayesR for milk production traits was equal to 6 and 3% for the bull only and the combined (bull and cow) reference datasets for Holstein cattle and about 5% for Jersey cattle. The largest increases in accuracy when using BayesR were observed for F% for both Holstein and Jersey cattle, probably because of the large-effect loci that segregate for this trait [7,8]. This occurs in spite of the apparent underestimation of phenotypic variance by BayesR reported in Table 3.

Genomic predictions for FERT (r ≈ 0.50) and SURV (r ≈ 0.43) in Holstein cattle were little affected by the prediction method or reference dataset used (see Additional file 3: Table S1). In Jersey cattle, accuracies obtained for FERT when using SNP information and only bulls for training were lower than the accuracy of pedigree-based predictions obtained when using the combined (bull and cow) reference dataset. Accuracies of genomic predictions for SURV in Jersey were rarely higher than those based on pedigree data. It is likely that these results for FERT and SURV reflect the low heritability and low accuracy of the records for these traits.

Adding polygenic effects to the prediction model increased the accuracy for milk production traits by on average 1 (Holstein) and 3% (Jersey) when using the combined (bull and cow) reference datasets (see Additional file 3: Table S1). However, adding polygenic effects increased bias by on average 13 and 17% in Holstein and Jersey cattle. The effect of adding polygenic effects on prediction bias was similar for the bull only and combined (bull and cow) reference datasets, and a similar bias was also observed in the pedigree (only) predictions. It seems that the pedigree relationships cause the increase in bias observed when polygenic effects are added to the genomic predictions, and this increase in bias was independent of whether the bull only or combined reference datasets were used (note that national genomic evaluations in ADHIS regress parent averages by approximately 0.6 to account for this bias). When predictions were based on SNP effects only, the overestimation of GEBV was greater in Jersey (average slope =0.94 for milk production traits) than in Holstein cattle (average slope =1.02) but, in general, the slope of the regressions did not differ notably from 1.

### Within- and multi-breed genomic predictions for Holstein and Jersey

Using the combined (bull and cow) reference datasets and excluding polygenic effects was found to give the ‘best’ (highest accuracy with least bias) genomic predictions for Holstein and Jersey validation animals. The observed accuracies and bias for these reference datasets when using only SNP effects for prediction are in Table 4 for milk production traits. These results show that BayesR resulted in an average increase in accuracy of 3 and 6% in the Holstein and Jersey single breed reference datasets, compared to GBLUP. There was little effect (±1%) of using the multi-breed reference dataset on prediction accuracies when using BayesR, and a small favourable effect (<2%) for GBLUP.

**Table 4:** **Accuracy and bias of within- and multi-breed genomic predictions for milk production traits**

**Ref** **dataset**	**Prediction method**	**Validation** **dataset**	**FY**		**MY**		**PY**		**F%**		**P%**		**Avg.**	
			**Acc.**	**Bias**	**Acc.**	**Bias**	**Acc.**	**Bias**	**Acc.**	**Bias**	**Acc.**	**Bias**	**Acc.**	**Bias**
*Prediction of Holstein*														
Holstein	*GBLUP*	Holstein	0.60	1.18	0.58	0.89	0.59	1.06	0.71	0.91	0.83	1.01	0.66	1.01
Holstein	*BayesR*	Holstein	0.63	1.22	0.62	0.89	0.58	1.02	0.81	1.01	0.83	1.02	0.69	1.03
Hol/Jer	*GBLUP*	Holstein	0.61	1.20	0.59	0.90	0.59	1.05	0.72	0.92	0.82	1.01	0.67	1.01
Hol/Jer	*BayesR*	Holstein	0.65	1.25	0.63	0.89	0.58	0.99	0.81	0.98	0.83	1.00	0.70	1.02
*Prediction of Jersey*														
Jersey	*GBLUP*	Jersey	0.56	0.88	0.62	0.93	0.67	1.20	0.63	0.83	0.75	0.88	0.65	0.95
Jersey	*BayesR*	Jersey	0.56	0.89	0.70	0.98	0.72	1.24	0.77	0.89	0.79	0.92	0.71	0.98
Hol/Jer	*GBLUP*	Jersey	0.58	0.88	0.64	0.91	0.69	1.17	0.66	0.82	0.77	0.90	0.67	0.94
Hol/Jer	*BayesR*	Jersey	0.56	0.93	0.69	0.95	0.71	1.18	0.76	0.92	0.79	0.87	0.70	0.97
Avg.	*GBLUP*		0.59	1.04	0.61	0.91	0.63	1.12	0.68	0.87	0.79	0.95	0.66	0.98
	*BayesR*		0.60	1.07	0.66	0.93	0.65	1.11	0.79	0.95	0.81	0.95	0.70	1.00

FY = fat yield (kg/lactation), MY = milk yield (L/lactation), PY = protein yield (kg/lactation), F% = fat percentage (%) and P% = protein percentage in milk (%); Acc. = accuracy, measured as *r*(**ŷ**, **y**), where **ŷ** is the prediction of genetic merit; Bias = bias of the prediction, measured as the regression coefficient, b (**ŷ**, **y**); standard errors are approximately $\frac{1}{\sqrt{262}}=0.062$ for the Holstein predictions, $\frac{1}{\sqrt{105}}=0.098$ for the Jersey predictions.

### Across-breed genomic predictions

Table 5 gives the accuracy and bias when prediction equations were tested in a breed not included in the reference population. Using predictions from the other breed resulted in a 40% reduction in prediction accuracy for the Holstein and Jersey breeds (Table 5), compared to prediction accuracies when the target breed was included in the reference dataset (Tables 4). The accuracy of prediction for the Australian Red breed was on average 3 and 9% greater when using a reference population that included both Jersey and Holstein animals compared to a reference population that included either Holstein or Jersey animals, respectively (Australian Red animals were never included in the reference population).

**Table 5:** **Accuracy and bias of across-breed genomic predictions for milk production traits**

**Ref datasets**	**Prediction method**	**Validation dataset**	**FY**		**MY**		**PY**		**F%**		**P%**		**Avg.**	
			**Acc.**	**Bias**	**Acc.**	**Bias**	**Acc.**	**Bias**	**Acc.**	**Bias**	**Acc.**	**Bias**	**Acc.**	**Bias**
*Prediction of Holstein or Jersey*														
Jersey	GBLUP	Holstein	0.09	0.55	0.10	0.54	0.09	0.63	0.15	0.42	0.17	0.48	0.12	0.51
Jersey	BayesR	Holstein	0.19	0.59	0.21	0.62	0.27	1.29	0.48	0.60	0.20	0.28	0.27	0.67
Holstein	GBLUP	Jersey	0.10	0.38	0.31	0.96	0.29	1.34	0.20	0.63	0.43	1.65	0.26	0.99
Holstein	BayesR	Jersey	0.09	0.21	0.30	0.53	0.26	0.71	0.33	0.56	0.42	0.79	0.28	0.56
*Prediction of Australian Reds*														
Holstein	GBLUP	AustRed	0.10	0.42	0.10	0.27	−0.01	0.00	0.41	0.94	0.48	1.25	0.22	0.57
Holstein	BayesR	AustRed	0.20	0.67	0.19	0.53	0.04	0.17	0.52	0.92	0.44	0.79	0.28	0.61
Jersey	GBLUP	AustRed	0.14	1.01	0.01	0.07	0.11	0.88	0.20	0.61	0.19	0.49	0.13	0.61
Jersey	BayesR	AustRed	0.35	1.60	0.08	0.28	0.19	1.12	0.41	0.59	0.21	0.33	0.25	0.78
Hol/Jer	GBLUP	AustRed	0.17	0.75	0.11	0.32	0.04	0.16	0.46	1.06	0.48	1.17	0.25	0.69
Hol/Jer	BayesR	AustRed	0.26	0.89	0.22	0.56	0.10	0.38	0.53	0.88	0.43	0.67	0.30	0.67
Avg.^1^	GBLUP		0.12	0.56	0.17	0.61	0.14	0.71	0.27	0.70	0.36	1.08	0.21	0.73
	BayesR		0.18	0.56	0.25	0.57	0.21	0.79	0.44	0.68	0.35	0.58	0.28	0.64

FY = fat yield (kg/lactation), MY = milk yield (L/lactation), PY = protein yield (kg/lactation), F% = fat percentage (%) and P% = protein percentage in milk (%); Acc. = accuracy, measured as *r*(**ŷ**, **y**), where **ŷ** is the prediction of genetic merit. Bias = bias of the prediction, measured as the regression coefficient, b (**ŷ**, **y**); standard errors are approximately $\frac{1}{\sqrt{262}}=0.062$ for the Holstein predictions, $\frac{1}{\sqrt{105}}=0.098$ for Jersey predictions, $\frac{1}{\sqrt{180}}=0.074$ for Australian Red predictions (average of the predictions for cow and bull validation sets; accuracies for each Australian Red bull and cow sets are in Additional file 3: Table S4 (see Additional file 3: Table S4); ^1^average across-breed prediction accuracy for GBLUP and BayesR is calculated using the average of the Australian Red predictions from the multi-breed Holstein/Jersey reference population, Jersey predictions from the Holstein reference population and Holstein predictions from the Jersey reference population.

Across-breed predictions showed an overall benefit of using BayesR compared to GBLUP (Table 5). Across various traits and breed combinations, BayesR outperformed GBLUP by on average 7% for all across-breed predictions in milk production traits. BayesR showed a very large (17%) advantage for F%, probably due to the segregation of SNPs with large effects [7,8] and a consistent advantage of 5 to 7% for FY, MY and PY. The combined effect of using BayesR and a multi-breed reference increased the accuracy of genomic predictions for the Australian Red animals by 8 and 17%, compared to the accuracies obtained from a single-breed reference dataset of Holstein or Jersey animals using GBLUP.

### Precision of QTL mapping with BayesR and GBLUP

We hypothesized that BayesR results in more accurate across-breed genomic predictions because it locates QTL effects more precisely in the genome. We examined the QTL regions identified by BayesR and GBLUP for QTL previously reported in the literature and identified several regions that contain QTL for milk production traits (e.g. *DGAT1* [19], *ABCG2* [35], *FASN* [36], *SCD* [37], the casein complex, *LALBA* and *PAEP (*formally *LGB)* [38]; *GHR* [39] and *AGPAT6* [40]). In most cases, except for *DGAT1* and *GHR*, the gene in the QTL region that was most over- (or under-) expressed in the mammary gland (compared to the 17 other tissues analysed) matched the loci reported in the literature (Table 6). Although *GHR* is cited as a candidate gene for the region identified on bovine chromosome BTA20, *CCL28* showed higher differential expression (P < 1 × 10^−29^) and it should be noted that this region is reported to contain other QTL [41].

**Table 6:** **Regions with large variance in local GEBV from BayesR for milk production traits**

**Gp**	**BTA**	**Window mid-point**		**Breed**	**Trait**					**Total loci**	Best candidate (mammary exp*)
		**Start**	**Stop**		**FY**	**MY**	**PY**	**F%**	**P%**		
FY-	5	93.375	94.075	H/J^3^	++	--	-	++	++	4	MGST1(+)
FY-	14	1.325	2.225	H/J^1,3^	++	--	--	++	++	70	DGAT1
FY-	27	36.075	36.375	H/J^3^	++	-	-	++		9	AGPAT6(+)
FY+	15	35.125	35.275	H/J	++		+			4	TPH1(−)
FY+	23	28.575	28.775	H/J	++	+	+		-	18	.
FY*n*	2	118.975	119.175	J				++	+	11	.
FY*n*	6	28.675	28.875	H	++					2	.
FY*n*	19	51.225	51.425	H/J	++			+		18	FASN(+)
FY*n*	26	21.025	21.225	H/J	++			+		15	SCD(+)
MY-	3	15.375	15.725	H/J^3^		+	-	-	--	33	MUC1(+)
MY-	11	104.125	104.325	H/J			-		--	25	ENTAG.12525(+)
MY+	1	144.325	144.525	H/J^3^	+	++	+	-	-	6	SLC37A1(+)
MY+	6	88.775	89.025	H		++	+	-	-	3	GC(−)
MY+	20	58.375	58.375	H/J	+				--	3	ANKH(+)
MY*n*	3	34.225	34.425	H/J					--	15	KIAA1324(+)
MY*n*	5	31.225	31.225	H					--	11	LALBA(+)
MY*n*	5	75.575	75.775	H/J^2,3^		++		-	--	11	CSF2RB(+)
MY*n*	5	118.175	118.375	H				-	--	2	.
MY*n*	6	37.475	38.725	H/J				-	--	19	ABCG2(+)
MY*n*	10	46.375	46.675	H/J^3^		+		-	--	6	.
MY*n*	12	70.225	70.275	J				-	--	1	ABCC4(−)
MY*n*	12	72.125	72.325	J				--	--	1	ENTAG.45751(+)
MY*n*	14	67.125	67.125	J					--	1	.
MY*n*	14	69.775	69.975	H		++		-	--	2	SDC2(+)
MY*n*	15	28.475	28.625	H					--	7	.
MY*n*	15	53.275	53.275	H		+			--	2	FCHSD2(+)
MY*n*	16	1.475	1.725	H/J		+		--	--	10	.
MY*n*	16	40.975	40.975	J					--	2	SUCO(+)
MY*n*	19	42.675	42.925	H/J				-	--	22	STAT5A(+)
MY*n*	19	61.075	61.225	H/J					--	2	KCNJ16(−)
MY*n*	20	29.225	32.125	H/J^3^		++		--	--	19	CCL28(+)/GHR(+)
MY*n*	20	34.425	34.625	H/J^3^		++		-	--	2	.
MY*n*	23	50.975	51.375	H/J		+			--	2	GMDS(+)
MY*n*	29	41.875	41.975	H					--	25	SLC3A2(+)
PY-	11	103.225	103.425	H/J	-	++	++	--		12	PAEP(+)
PY+	5	75.075	75.275	H/J^2,3^	+	++	++			11	ENTAG.38652(+)
PY+	5	88.725	89.025	H/J	+	++	++	-		8	GYS2(−)
PY+	6	87.025	87.525	H/J^1,3^		+	++		++	14	CSN1S1(+)
PY+	10	16.725	16.925	H	+	+	++			2	TLE3(+)
PY+	16	31.025	31.025	H	+	+	++			3	.
PY+	18	18.325	18.425	J	+	+	++			3	.
PY+	23	39.175	39.375	J	+	+	++			8	KIF13A(+)
PY+	28	18.575	18.775	H/J		++	++			3	.

FY = fat yield (kg/lactation), MY = milk yield (L/lactation), PY = protein yield (kg/lactation), F% = fat percentage (%) and P% = protein percentage in milk (%); ++ or -- indicates that the largest effect of a window in a region was greater than 50 times that of an average window and + or – indicates that window effects are greater than 3 times the average; directions of pleiotropic effects were determined by the correlation of GEBV between traits; regions are H or J (only) QTL when trait effects were greater than 50 times the mean in the alternate breed; descriptions of the identified genes with differential expression are in Additional file 3: Table S5 (see Additional file 3: Table S5). ^*^over- (+) or under- (−) expression in mammary tissue (P < 1 × 10^−5^) relative to 17 other tissue types. ^1^some ambiguity in the QTL effects and pattern of effects, possibly indicate > 1 QTL or alleles. ^2^this region had two clear patterns of QTL effects and was split into two regions. ^3^similar QTL region also identified by GBLUP.

**Figure 2:**
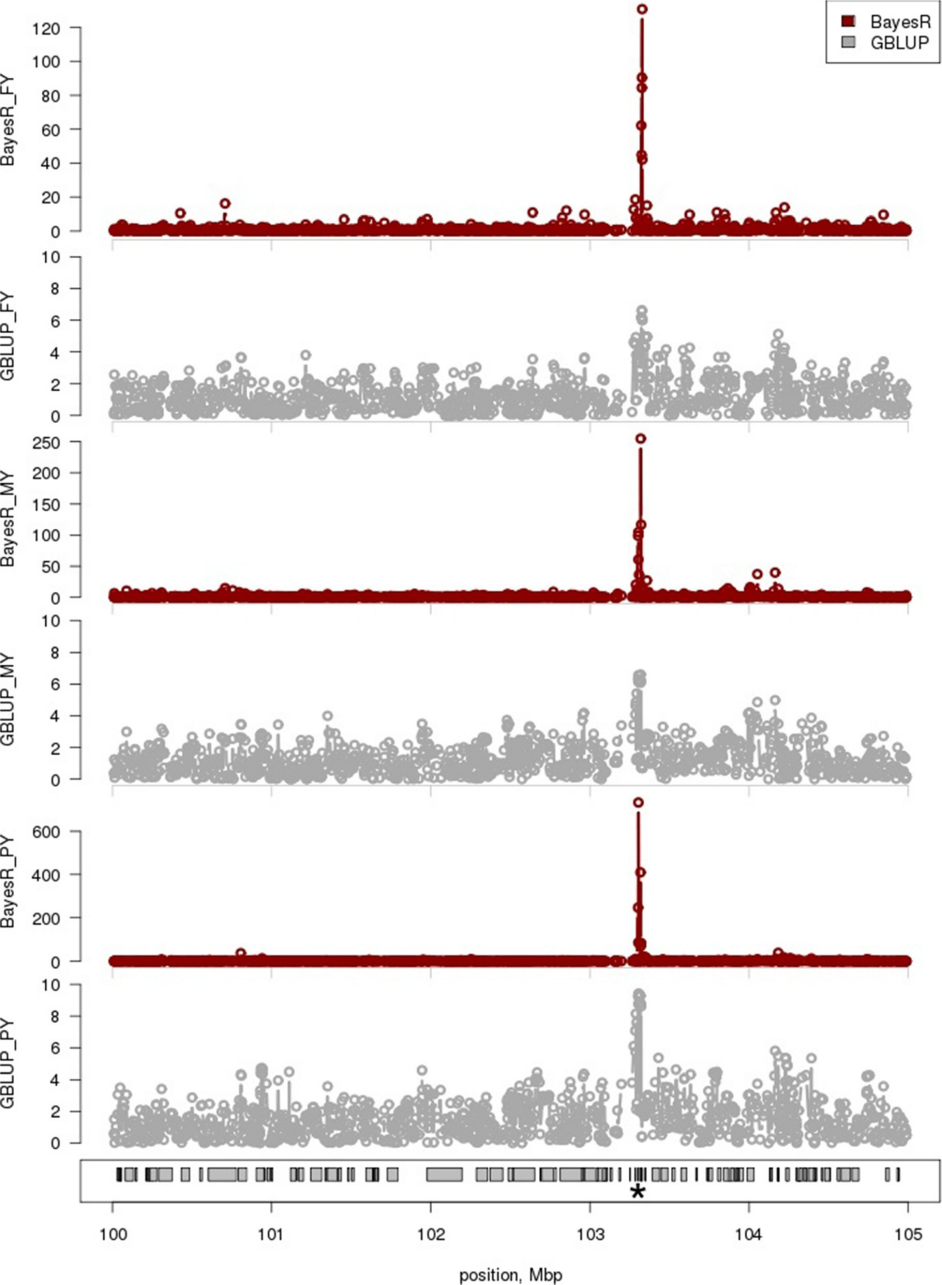
**SNP effects estimated by BayesR and GBLUP for FY, MY and PY near the** ***PAEP*** **gene.** Shown is the (mean corrected) absolute value of SNP effect estimates from the bull and cow, multi-breed reference population. Traits are FY = fat yield, MY = milk yield and PY = protein yield. The position of *PAEP* on BTA11 is marked (*). Note the changed y-axis scale for each graph.

**Figure 3:**
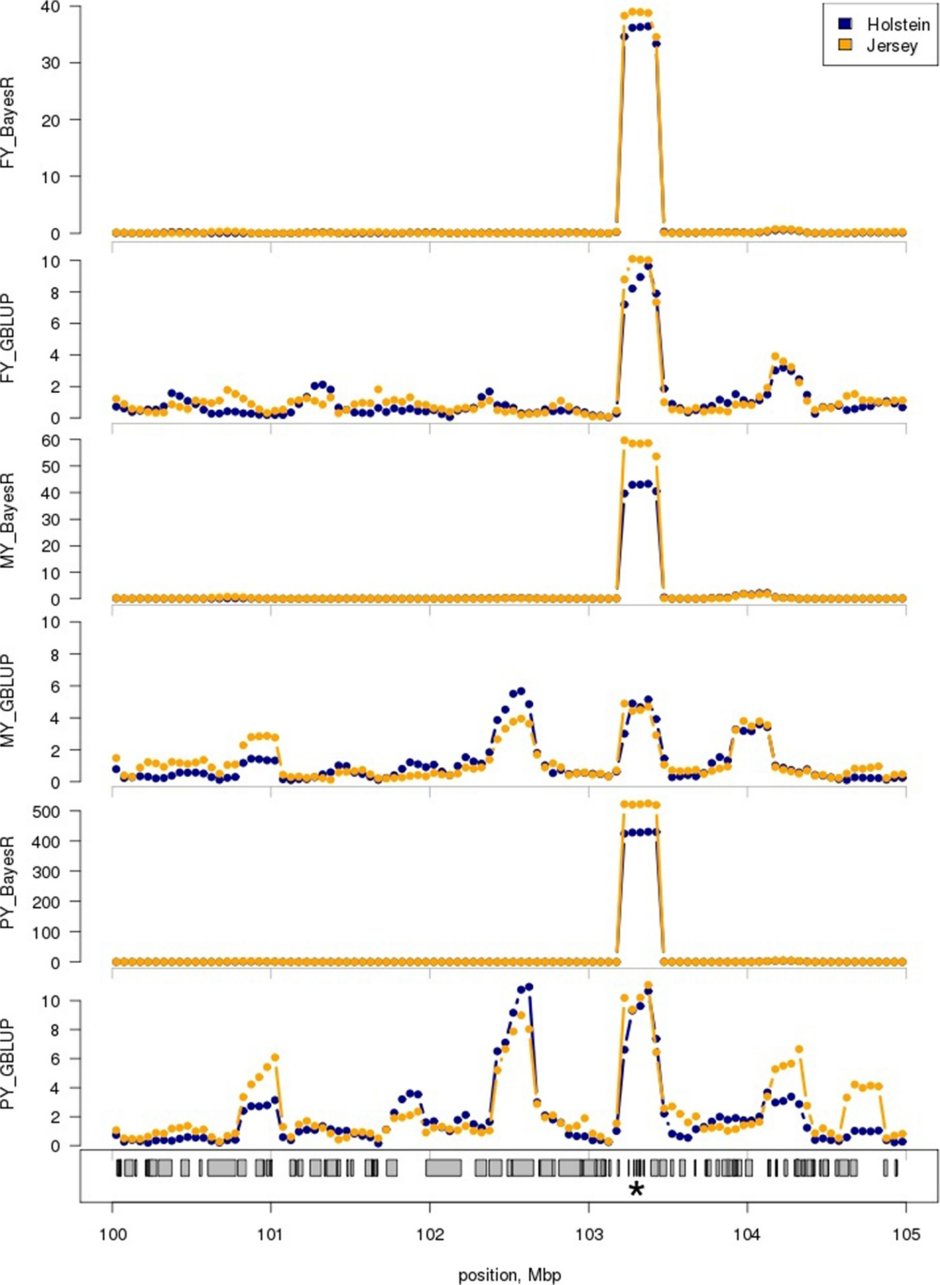
**Local GEBV variance near the** ***PAEP*** **gene for FY, MY and PY using BayesR and GBLUP** ***.*** Shown is the (mean corrected) GEBV variance in 250 kb windows for Holstein and Jersey reference animals from SNP effects estimated from the bull and cow, multi-breed reference population. Traits are FY = fat yield, MY = milk yield and PY = protein yield. The position of *PAEP* on BTA11 is marked (*). Note the changed y-axis scale for each graph.

To investigate the precision of GBLUP versus BayesR in mapping QTL, we specifically investigated the mapping of the *PAEP* gene. Figure 2 shows the absolute value of the SNP effect estimates in the region. With BayesR, SNPs were identified that have larger effects on milk production traits than most of the surrounding SNPs. In contrast, with GBLUP all the SNPs in the identified region had small effects, although there was possibly a small increase in SNP effect estimates for SNPs for which BayesR also found larger effects. In spite of these small effects, somewhat surprisingly, the local variance in GEBV using GBLUP did find peaks in the region of *PAEP* (Figure 3). This is probably due to the SNP estimates in the linear combination for local GEBV reinforcing each other in the area of the peak but almost cancelling each other out in other regions. However, a careful inspection showed that, although GBLUP often indicated a region with large GEBV variance near the QTL, the maximum variance was larger and more concentrated near the reported QTL for BayesR than for GBLUP. This is due to the heterogeneous variance assumptions in the BayesR method*,* which allow SNPs in high LD with the QTL to have larger effects.

*PAEP* is reported to have a large effect on PY and smaller effects on MY and FY, and encodes the primary whey protein of bovine milk [38]. Although GBLUP indicates a region of high GEBV variance that encompasses *PAEP*, BayesR captured this pattern of effects more accurately and estimated appropriately the SNPs with large effects near *PAEP* in the analysis of PY. The analysis of FY, MY and PY with GBLUP likely captured the effect of *PAEP,* but the effect seemed to be dispersed over a large region that covered possibly the entire 5 Mbp region shown in Figure 2.

A second example of QTL mapping is provided in Figures S2 and S3 (see Additional file 1: Figures S2 and S3) for *AGPAT6*. In agreement with the other reports [40], we observed *AGPAT6* to have a large effect on FY with smaller effects on MY and PY. Similar to *PAEP*, the effect that was estimated for *AGPAT6* by GBLUP was spread over a larger genomic region than the effect that was estimated by BayesR. Interestingly, the effects of *AGPAT6* on PY estimated by both methods were very similar. It seems that the difference between BayesR and GBLUP declines as the effect size of a locus decreases. In addition, there may be two other QTL near *AGPAT6* that affect MY and PY.

All QTL with large effects (defined by a local GEBV variance greater than 50 times that of an average window) identified in the BayesR analysis with the multi-breed bull and cow reference dataset and their pleiotropic effects are presented in Table 6. Several of these regions were also identified by GBLUP and by a previous study on this data using a genome-wide association approach with single-marker regression analysis [18]. We identified QTL from eight of the nine possible groupings for pleiotropic effects. That is, large-effect QTL for milk yield traits could have positive, negative or no (observable) correlation with other milk yield traits.

## Discussion

The accuracy of genomic predictions using GBLUP depends on the size of the reference population [42,43]. Thus, when a large reference population is available for a single breed of dairy cattle, such as Holstein, GBLUP captures most of the potential accuracy for genomic predictions and there seems little benefit in using nonlinear methods for prediction, such as BayesR, or high-density genomic markers [9]. However, when predictions need to be more robust and are used to predict genetic merit of distantly-related animals, such as animals in future generations or animals from different breeds, the benefits of genomic prediction using GBLUP with medium-density SNPs decreases compared to nonlinear methods. This was first pointed out by Habier *et al*. [1] who reported poor predictions with GBLUP over successive generations compared to BayesB. Here, we show the advantages of nonlinear genomic prediction methods with across-breed predictions. We showed that the accuracy of genomic predictions obtained using BayesR increased by 8 and 17% compared to GBLUP predictions from a single breed when they were estimated for Australian Red animals from a multi-breed Holstein/Jersey reference population. In regions that contain known mutations that affect milk production, we demonstrated that BayesR localises SNP effects to smaller genomic regions than GBLUP. Thus, robust genomic prediction of genetic merit and QTL mapping are related problems, which can both be accomplished by nonlinear methods such as BayesR.

Increasing the size of the reference population by including cows increased accuracy of genomic predictions by 4.2 to 5.4% for traits with moderate to high heritability. Adding cows had little or no effect on the bias of predictions. This is in contrast to the bias introduced when adding cows in French and US studies [44,45], possibly because, in our case, cows were sampled from commercial herds with little potential for bias from preferential treatment and animals were not selectively genotyped based on genetic merit. In our data, adding cows benefitted predictions despite their phenotypic records being less accurate than records on bulls. This is probably because the size of the reference population increased substantially by adding the cows, by almost three times in the Holstein data and five times in the Jersey data. A further benefit of using the Holstein cows was that they were more genetically diverse than the Holstein bull population (see Additional file 1: Figure S4). This diversity is useful to identify SNPs that track causative mutations, and thus contributes to improving the robustness of genomic predictions. Since the cows that were added to the reference population were animals from commercial farms, it is possible that some animals may have been recently admixed with another breed and present varying degrees of traditional ancestry with Australian dairy cattle, such as British Friesians.

The BayesR QTL mapping approach, combined with expression data from mammary gland tissue, was powerful for the identification of many previously reported QTL that are known to be involved in milk production. For the known QTL, the patterns of pleiotropic effects estimated by BayesR matched the reported effects for some mutations. This study suggests that QTL mapping using a nonlinear approach and considering multiple traits may improve the mapping precision. This will be most beneficial for QTL with large effects on one trait and smaller effects on another trait. For example, the large effect of *AGPAT6* on FY could help to more precisely map the smaller effects of this locus on PY. We observed little difference between GBLUP and BayesR in the across-breed prediction for P%, presumably because it is controlled by many QTL with small effects. A strategy that uses multiple traits to assist the localisation of QTL may be useful to increase robustness and accuracy of across-breed predictions for traits such as P%.

Our analysis identified several interesting candidate loci for milk production traits that (1) were identified as QTL with both BayesR and GBLUP, (2) were highly over- (or under-) expressed in the mammary gland compared to the other 17 tissue types analysed and (3) have functions in milk synthesis that have been described independently. It may be interesting to further study these loci, which include: *SLC37A1* that encodes a glucose-6-phosphate transporter involved in the homeostasis of blood glucose [46]; *MUC1* that encodes a glycoprotein that is a component of the surface membrane of fat globules in milk [47] and is also assumed to contribute to epithelial cell defence against bacteria; and *CSF2RB,* which is involved in the JAK-STAT signalling pathway (the JAK-STAT pathway has a central role in prolactin signalling [48]). Another promising novel candidate gene is *TPH1*, which is involved in mammary gland development (GO:0067074) and serotonin synthesis [49].

## Conclusions

The use of a nonlinear method (such as BayesR) and high-density SNP genotypes, combined with a multi-breed reference population that included cows and bulls, led to the highest accuracies of genomic prediction, especially for a breed that was not included in the reference population. The advantage of BayesR over GBLUP is due to its better use of SNPs that are close to the causal mutation. Thus, the accuracy of GEBV derived using BayesR should be greater than that of GEBV derived using GBLUP for a variety of target populations and across multiple generations. It seems that BayesR is a useful methodology to map genes responsible for variation in quantitative traits.

## Additional files

Additional file 1: Figure S1. Linkage disequilibrium in Holstein, Jersey and Australian Red dairy cattle. Figure S1. Shows the average r^2^ (correlation) between SNP pairs and the average distance between adjacent SNPs (vertical grey line) on BTA1. **Figure S2.** SNP effects estimated by BayesR and GBLUP for FY, MY and PY near the gene AGPAT6. **Figure S2.** shows the (mean corrected) absolute SNP effects for Holstein and Jersey animals from the bull and cow, multi-breed reference population. Traits are FY = fat yield, MY = milk yield and PY = protein yield. The position of *AGPAT6* on BTA27 is marked (*). Note the y-axis scale is changed for each graph. **Figure S3.** Local GEBV variance near the gene AGPAT6 for FY, MY and PY using BayesR and GBLUP. Figure S3 shows the (mean corrected) GEBV variance in 250 kb windows for Holstein and Jersey animals from the bull and cow, multi-breed reference population. Traits are FY = fat yield, MY = milk yield and PY = protein yield. The position of the gene *AGPAT6* on BTA27 is marked (*). Note the y-axis scale is changed for each graph. **Figure S4.** Relationship between Holstein and Jersey cows and bulls in the reference and validation datasets. **Figure S4.** shows the principle components from the **G** matrix using the eigen() function in R [50]. The named subset (i.e. Holstein cows, top left) is highlighted in blue in each panel. Additional file 2:
**Derivation of the full conditional likelihood function.** Additional file 2 provides the details for the derivation of the full conditional likelihood function. Additional file 3:
**Effect of reference dataset, prediction method and polygenic term on genomic predictions of milk traits, stature, fertility and survival.** **Table S1**. Bold characters show the prediction accuracies (Acc.) and biases using only pedigree information or GBLUP with bull only or bull and cow reference datasets. Below the numbers in bold characters are the average effect on the prediction when changing of the method used to predict SNP effects (+BayesR), adding non-target breed animals to the reference (i.e. multi-breed reference, e.g. +Holstein) and adding the polygenic effect (+polygenic). These estimates are from the linear model fitted to the prediction results and effects are deviations from the GBLUP predictions. **Table S2.** Genomic prediction accuracy and bias for the Holstein validation dataset. Genomic predictions accuracy and bias for the Holstein validation population using different combinations of reference datasets (bulls or bulls and cows), method of prediction (GBLUP or BayesR), breed composition in the reference population (single or both breeds), and with or without inclusion of the polygenic term in the prediction. **Table S3.** Genomic prediction accuracy and bias for the Jersey validation dataset. Genomic predictions accuracy and bias for the Jersey validation population using different combinations of reference datasets (bulls or bulls and cows), method of prediction (GBLUP or BayesR), breed composition in the reference population (single or both breeds), and with or without inclusion of the polygenic term in the prediction. **Table S4.** Across-breed prediction accuracies for Australian Red animals. Prediction accuracies and bias for genomic predictions in Australian Red animals are provided separately for the bull and cow validation sets. **Table S5.** Details on the genes identified from the mammary expression data and listed in **Table** 6 **.** Tables S5 gives the Ensebl gene ID, full name, chromosome, gene start and end on the bovine genome (bp) and the description of the genes listed in **Table** 6 **.**
