# Supplementary material for: Improved precision of QTL mapping using a nonlinear Bayesian method in a multi-breed population leads to greater accuracy of across-breed genomic predictions
Source: Genet Sel Evol. 2015 Apr 17;47(1):29. doi: 10.1186/s12711-014-0074-4 (PMC4399226; doi:10.1186/s12711-014-0074-4)
Supplement: Additional file 1: Figure S1. — Linkage disequilibrium in Holstein, Jersey and Australian Red dairy cattle. Figure S1. Shows the average r2 (correlation) between SNP pairs and the average distance between adjacent SNPs (vertical grey line) on BTA1. Figure S2. SNP effects estimated by BayesR and GBLUP for FY, MY and PY near the gene AGPAT6. Figure S2. shows the (mean corrected) absolute SNP effects for Holstein and Jersey animals from the bull and cow, multi-breed reference population. Traits are FY = fat yield, MY = milk yield and PY = protein yield. The position of AGPAT6 on BTA27 is marked (*). Note the y-axis scale is changed for each graph. Figure S3. Local GEBV variance near the gene AGPAT6 for FY, MY and PY using BayesR and GBLUP. Figure S3 shows the (mean corrected) GEBV variance in 250 kb windows for Holstein and Jersey animals from the bull and cow, multi-breed reference population. Traits are FY = fat yield, MY = milk yield and PY = protein yield. The position of the gene AGPAT6 on BTA27 is marked (*). Note the y-axis scale is changed for each graph. Figure S4. Relationship between Holstein and Jersey cows and bulls in the reference and validation datasets. Figure S4. shows the principle components from the G matrix using the eigen() function in R [50]. The named subset (i.e. Holstein cows, top left) is highlighted in blue in each panel. [file 12711_2014_74_MOESM1_ESM.pdf]

Additional file 1

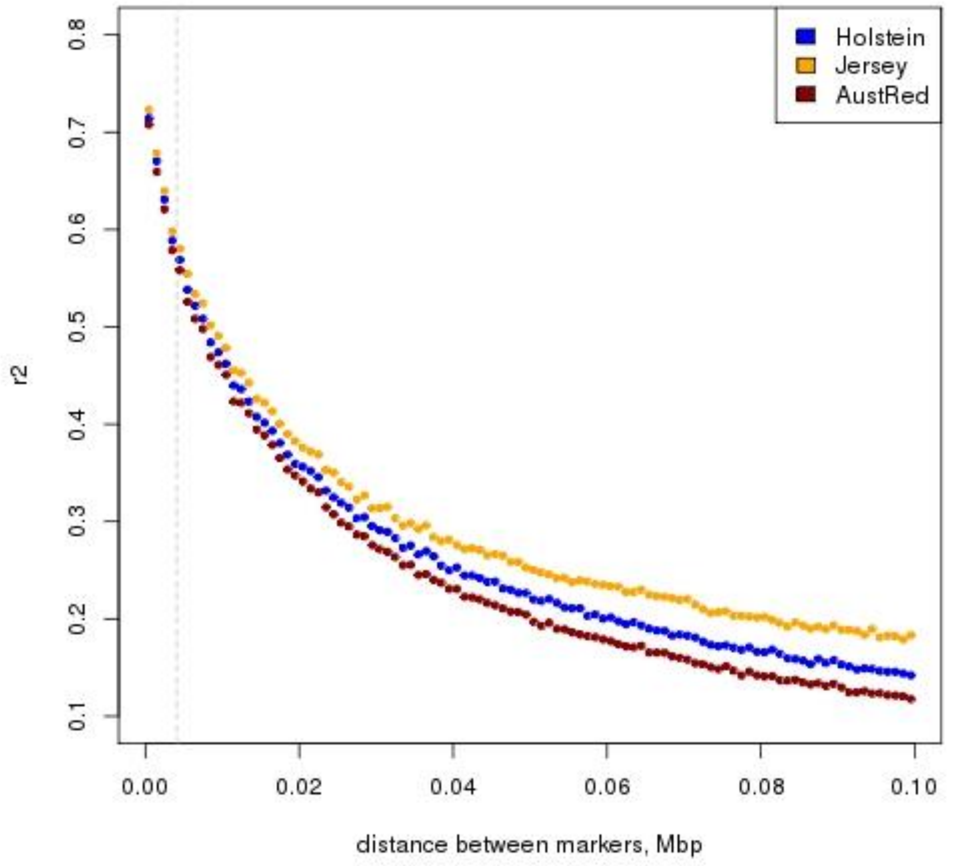

Figure S1 Linkage disequilibrium in Holstein, Jersey and Australian Red dairy cattle

Shown is the average r^2 (correlation) between SNP pairs and the average distance between adjacent SNP (vertical grey line) on BTA1.

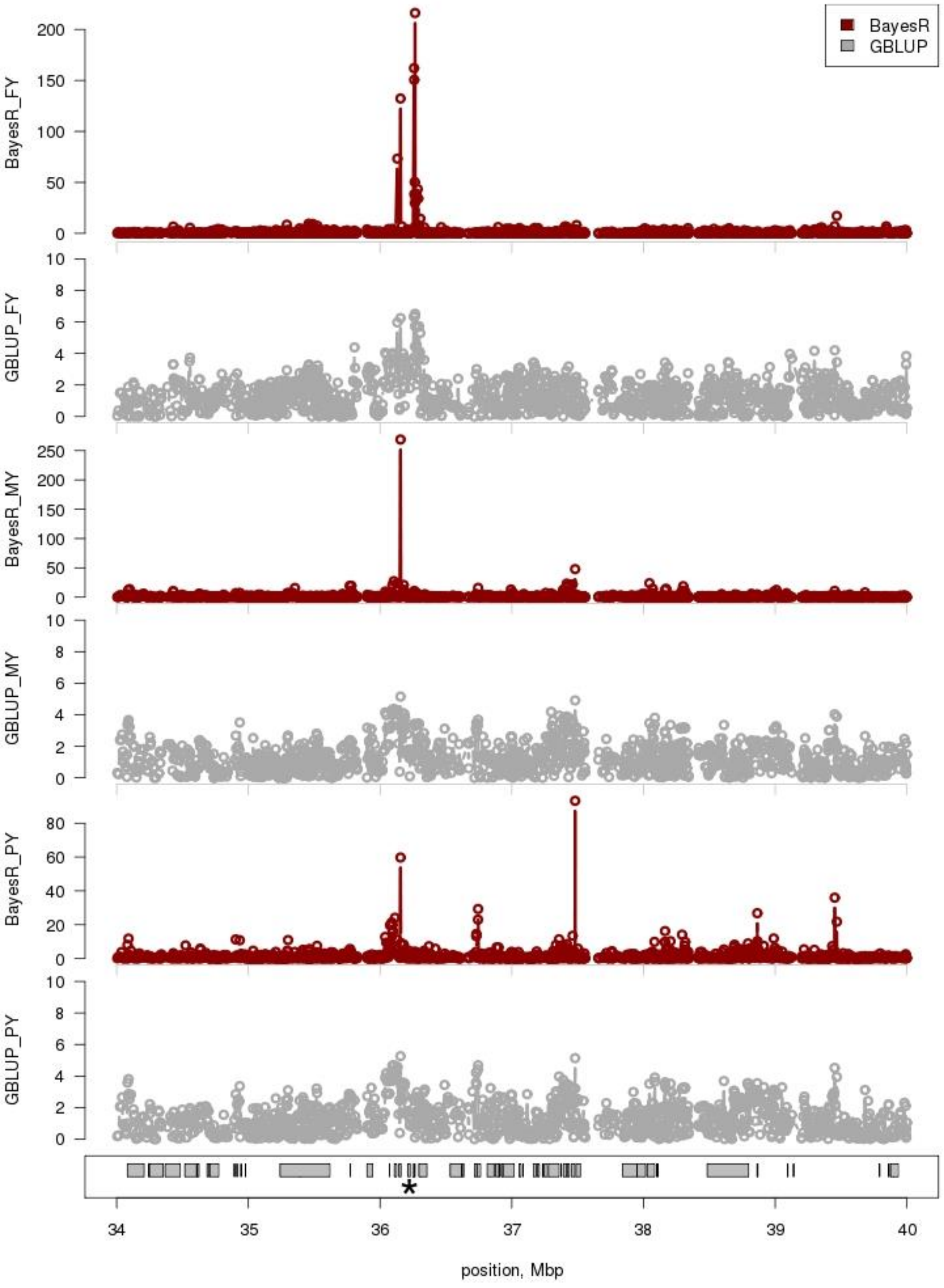

Figure S2 SNP effects estimated by BayesR and GBLUP for FY, MY and PY near AGPAT6

Shown is the (mean corrected) absolute SNP effect for Holstein and Jersey animals from the bull and cow, multi-breed reference population. Traits are FY = fat yield, MY = milk yield and PY = protein yield. The position of AGPAT6 on BTA27 is marked (*). Note the changed y-axis scale for each graph.

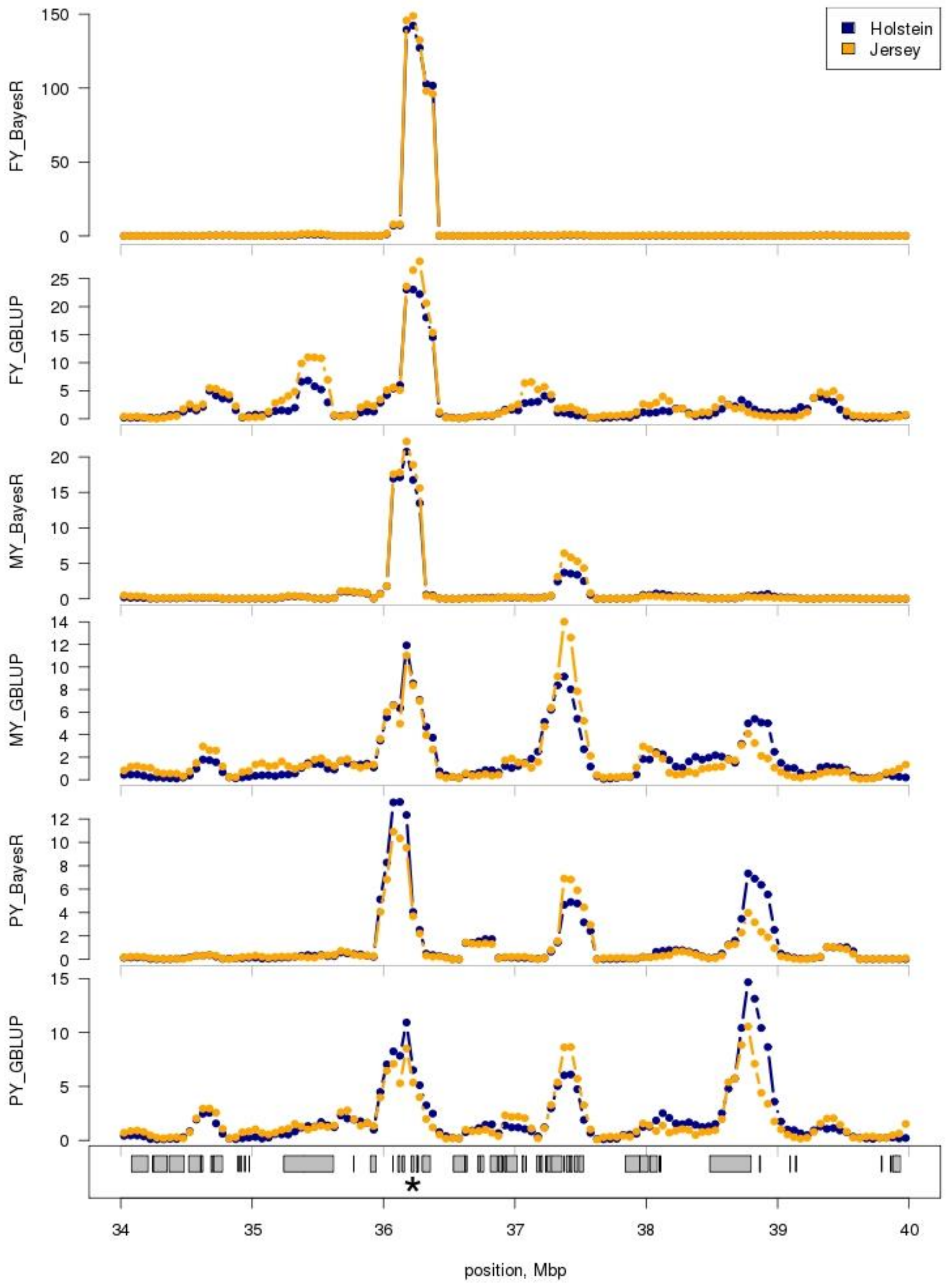

Figure S3 Local GEBV variance near AGPAT6 for FY, MY and PY using BayesR and GBLUP

Shown is the (mean corrected) GEBV variance in 250kb windows for Holstein and Jersey animals from the bull and cow, multi-breed reference population. Traits are FY = fat yield, MY = milk yield and PY = protein yield. The position of AGPAT6 on BTA27 is marked (*). Note the changed y-axis scale for each graph

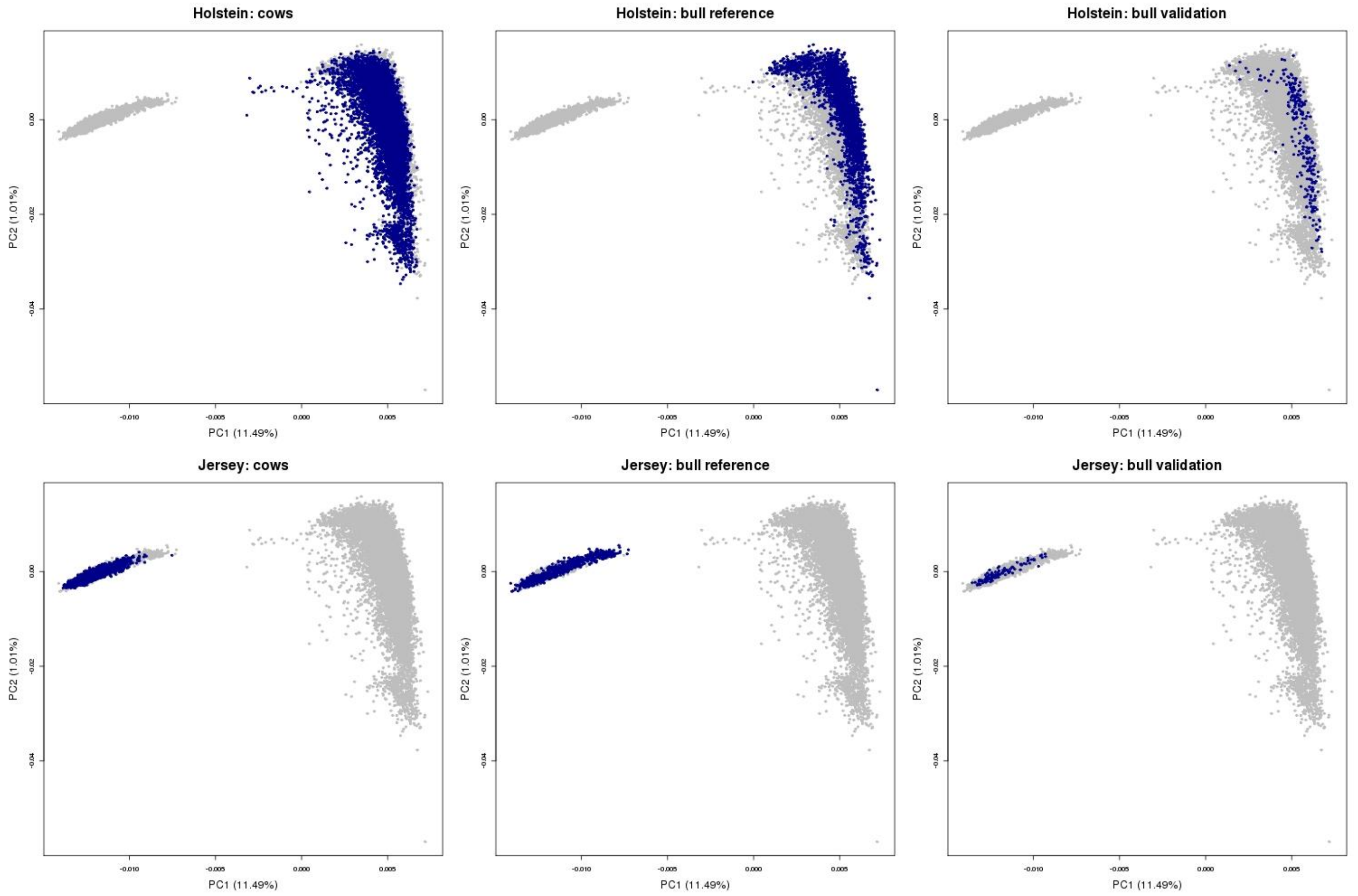

Figure S4 Relationship between Holstein and Jersey cows and bulls in the reference and validation datasets

Principle components from the G matrix using the eigen() function in R [51]. The named subset (i.e. Holstein cows, top left) is highlighted in blue for each panel.
