# Supplementary material for: Improved precision of QTL mapping using a nonlinear Bayesian method in a multi-breed population leads to greater accuracy of across-breed genomic predictions
Source: Genet Sel Evol. 2015 Apr 17;47(1):29. doi: 10.1186/s12711-014-0074-4 (PMC4399226; doi:10.1186/s12711-014-0074-4)
Supplement: Additional file 2: — Derivation of the full conditional likelihood function. Additional file 2 provides the details for the derivation of the full conditional likelihood function. [file 12711_2014_74_MOESM2_ESM.pdf]

Additional file 2

Derivation of the full conditional likelihood function

The likelihood that marker *j* is from distribution *k* is

$$\mathrm{L}\left(v_{j,k}|\sigma_k^2\right) = -0.5[\ln(2\pi) + \ln(|\mathbf{V}|) + \mathbf{y}_j^{*\prime}\mathbf{V}^{-1}\mathbf{y}_j^*]$$

where $\mathbf{V}$ is the (residual) phenotypic variance-covariance matrix and $\mathbf{y}^{*\prime}\mathbf{V}^{-1}\mathbf{y}^*$ simplifies to $\mathbf{y}_j^{*\prime}\mathbf{R}^{-1}\mathbf{y}_j^* - \mathbf{y}_j^{*\prime}\mathbf{R}^{-1}\mathbf{W}_{.j}v_{j,k}$ (i.e. where $v_{j,k} = [\mathbf{W}_{.j}{}'\mathbf{R}^{-1}\mathbf{W}_{.j} + \sigma_k^{-2}]^{-1}\mathbf{W}_{.j}{}'\mathbf{R}^{-1}\mathbf{y}^*$, following eq. [vi] in the main paper). Since $\mathrm{V} = \mathbf{W}_{.j}\mathbf{W}_{.j}{}'\sigma_k^2 + \mathbf{R}$, then using Sylvester's theorem $\ln(|\mathbf{V}|) = \ln(|\mathbf{R}|) + \ln(|\mathbf{I} + \mathbf{W}_{.j}{}'\mathbf{R}^{-1}\mathbf{W}_{.j}\sigma_k^2|)$ (see PART A below) and because In(|**R**|) is constant over any σ^2_k, then the inverse phenotypic variance-covariance matrix is $\mathbf{V}^{-1} = \mathbf{R}^{-1} - \mathbf{R}^{-1}\mathbf{W}_{.j}[\mathbf{W}_{.j}{}'\mathbf{R}^{-1}\mathbf{W}_{.j} + \sigma_k^{-2}]^{-1}\mathbf{W}_{.j}{}'\mathbf{R}^{-1}$ (see PART B for proof of $\mathbf{V}\mathbf{V}^{-1} = \mathbf{I}$). Hence the full conditional likelihood is:

$$\mathrm{L}\left(v_{j,k}|\sigma_k^2\right) = -0.5[\ln\left(1 + \mathbf{W}_{.j}{}'\mathbf{R}^{-1}\mathbf{W}_{.j}\sigma_k^2\right) + \mathbf{y}_j^{*\prime}\mathbf{R}^{-1}\mathbf{y}_j^* - \mathbf{y}_j^{*\prime}\mathbf{R}^{-1}\mathbf{W}_{.j}v_{j,k}] + \ln(pr_k),$$

where pr_k is the current estimate for the proportion of markers from distribution *k*.

PART A:

Sylvester's theorem states that:

$$|\mathbf{I}_n + \mathbf{AB}| = |\mathbf{I}_m + \mathbf{BA}|$$

where $\mathbf{I}_n$ is an *n* by *n* identity matrix, **A** is an *n* by *m* matrix and **B** is a *m* by *n* matrix. By extension, if **X** is a *n* by *n* square matrix then

$$|\mathbf{X} + \mathbf{AB}| = |\mathbf{X}|\left|\mathbf{I}_n + \mathbf{X}^{-1}\mathbf{AB}\right|$$
$$= |\mathbf{X}|\left|\mathbf{I}_m + \mathbf{B}\mathbf{X}^{-1}\mathbf{A}\right|$$

Similarly, if

$$|\mathbf{V}| = |\mathbf{W}\mathbf{W}'\sigma_k^2 + \mathbf{R}|$$
$$= |\mathbf{R}|\left|\mathbf{I} + \mathbf{R}^{-1}\mathbf{W}\mathbf{W}'\sigma_k^2\right|$$
$$= |\mathbf{R}|\left|\mathbf{I} + \mathbf{W}'\mathbf{R}^{-1}\mathbf{W}\sigma_k^2\right|$$

PART B:

Proof that $\mathbf{V}\mathbf{V}^{-1} = \mathbf{I}$ [following 27]:

Let

$$\mathbf{Q} = \left[\mathbf{W}'_{.j}\mathbf{R}^{-1}\mathbf{W}_{.j} + \sigma_k^{-2}\right]^{-1}$$

$\mathbf{V}\mathbf{V}^{-1}$

$$= \left[\mathbf{R} + \mathbf{W}_{.j}\mathbf{W}'_{.j}\sigma_k^2\right]\left[\mathbf{R}^{-1} + \mathbf{R}^{-1}\mathbf{W}_{.j}\mathbf{Q}\mathbf{W}'_{.j}\mathbf{R}^{-1}\right]$$
$$= \mathbf{I} + \mathbf{W}_{.j}\mathbf{W}'_{.j}\mathbf{R}^{-1}\sigma_k^2 - \mathbf{W}_{.j}\mathbf{Q}\mathbf{W}'_{.j}\mathbf{R}^{-1} - \mathbf{W}_{.j}\mathbf{W}'_{.j}\mathbf{R}^{-1}\mathbf{W}_{.j}\mathbf{Q}\mathbf{W}'_{.j}\mathbf{R}^{-1}\sigma_k^2$$
$$= \mathbf{I} + \mathbf{W}_{.j}\mathbf{W}'_{.j}\mathbf{R}^{-1}\sigma_k^2 - \mathbf{W}_{.j}[\mathbf{I} + \mathbf{W}'_{.j}\mathbf{R}^{-1}\mathbf{W}_{.j}\sigma_k^2]\mathbf{Q}\mathbf{W}'_{.j}\mathbf{R}^{-1}$$
$$= \mathbf{I} + \mathbf{W}_{.j}\mathbf{W}'_{.j}\mathbf{R}^{-1}\sigma_k^2 - \mathbf{W}_{.j}\sigma_k^2[\sigma_k^{-2} + \mathbf{W}'_{.j}\mathbf{R}^{-1}\mathbf{W}_{.j}]\mathbf{Q}\mathbf{W}'_{.j}\mathbf{R}^{-1}$$
$$= \mathbf{I} + \mathbf{W}_{.j}\mathbf{W}'_{.j}\mathbf{R}^{-1}\sigma_k^2 - \mathbf{W}_{.j}\mathbf{Q}^{-1}\mathbf{Q}\mathbf{W}'_{.j}\mathbf{R}^{-1}$$
$$= \mathbf{I}$$
